# MR Neuroimaging in Pediatric Inborn Errors of Metabolism

**DOI:** 10.3390/diagnostics12040861

**Published:** 2022-03-30

**Authors:** Lillian M. Lai, Andrea L. Gropman, Matthew T. Whitehead

**Affiliations:** 1 Department of Radiology, University of Iowa Hospitals and Clinics, Iowa City, IA 52242, USA; lmlai@healthcare.uiowa.edu; 2 Department of Radiology, Children’s Hospital Los Angeles, Los Angeles, CA 90027, USA; 3 Department of Neurology, Children’s National, Washington, DC 20010, USA; agropman@childrensnational.org; 4 Department of Radiology, Children’s National, Washington, DC 20010, USA

**Keywords:** inborn, errors, metabolism, MRI, MRS, magnetic, resonance, spectroscopy

## Abstract

Inborn errors of metabolism (IEM) are a group of disorders due to functional defects in one or more metabolic pathways that can cause considerable morbidity and death if not diagnosed early. While individually rare, the estimated global prevalence of IEMs comprises a substantial number of neonatal and infantile disorders affecting the central nervous system. Clinical manifestations of IEMs may be nonspecific. Newborn metabolic screens do not capture all IEMs, and likewise, genetic testing may not always detect pathogenic variants. Neuroimaging is a critical component of the work-up, given that imaging sometimes occurs before prenatal screen results are available, which may allow for recognition of imaging patterns that lead to early diagnosis and treatment of IEMs. This review will demonstrate the role of magnetic resonance imaging (MRI) and proton magnetic resonance spectroscopy (^1^H MRS) in the evaluation of IEMs. The focus will be on scenarios where MRI and ^1^H MRS are suggestive of or diagnostic for IEMs, or alternatively, refute the diagnosis.

## 1. Introduction

Inborn errors of metabolism (IEM) are a group of disorders caused by a defect or defects in the functioning of one or more intermediate metabolic pathways, either due to deficiencies or superactivities of enzymes or transporters, chaperone deficiencies, or transcription factor deficits [1]. Prompt diagnosis is important to guide therapeutic measures and reduce or prevent morbidity and death. While individually rare, the estimated global prevalence of all cause IEMs is thought to range from approximately 1 in 800 to 2500 live births [1,2]. The most prevalent disorders (ordered most to least prevalent) include amino acid, lysosomal storage, organic acid, mitochondrial, fatty acid, carbohydrate metabolism, peroxisomal, and urea cycle disorders [2]. 

Neuroimaging plays an important role in diagnosis and treatment monitoring [3,4,5,6]. Many IEMs present in the neonatal or early infantile period [7]. Newborn metabolic screens do not capture all IEMs, both false negatives and false positive results can occur, and genetic testing may not always detect pathogenic mutations. Imaging is sometimes performed before the newborn screen results are available, and when positive and correctly interpreted, provides a chance for early intervention. Since patients with IEMs are more likely to have their initial imaging at smaller community hospitals, it is important that all radiologists that interpret neuroimaging be familiar with findings that may suggest an IEM such that patients can be swiftly plugged into the appropriate therapeutic algorithm or transferred to a specialty center.

It would be impossible to cover more than a minority of the over 1000 currently known IEMs [1]. We will limit the scope of this review to clarify the role of magnetic resonance imaging (MRI) and proton magnetic resonance spectroscopy (^1^H MRS) in the evaluation of IEMs. We will focus on cases where MRI and ^1^H MRS patterns are suggestive, diagnostic, or refutative of a diagnosis in both specific and nonspecific clinical scenarios.

## 2. Clinical Scenarios Suggestive of IEM and General Classification

Most IEM-related symptoms are nonspecific in the neonatal period (e.g., encephalopathy, metabolic acidosis, hypoglycemia, cardiac or liver disorders) and can be mimicked by hypoxic ischemic encephalopathy (HIE), sepsis and congenital heart disease. To make matters more complicated, patients with IEMs may also present with cardiac disease, sepsis, and/or hypoglycemia.

However, certain clinical scenarios may suggest IEM, and would warrant an MRI and MRS as soon as feasible. Usually, these include a history of normal birth/delivery, a symptom free interval, followed by an unexplained clinical decline, especially for disorders resulting in build-up of toxic metabolites or molecules. Other important clinical clues include multisystemic abnormalities, intellectual disabilities, seizure(s) under 6 months of age, prolonged instability, or progression of symptoms (conversely, HIE stabilizes by 2–3 weeks). Any history of metabolic diseases within the family, consanguinity, multiple miscarriages, and/or unexplained neonatal deaths also raise the possibility of an IEM [8]. Ultimately, clinical manifestations of IEMs are based on the effects of functional deficits, which can help with classification into groups: (1) intoxication disorders, (2) disorders of biosynthesis and breakdown of complex molecules, and (3) energy production disorders [7]. Appendix Table A1 summarizes the key MRI/MRS features of diagnosable IEMs within these general categories.

### 2.1. Intoxication Disorder (e.g., Amino Acid Metabolism, Urea Cycle, and Organic Acid Disorders)

These are characterized by a history of normal birth/delivery, variable symptom-free interval followed by clinical decline with acute/chronic intoxication of the central nervous system (CNS) due to accumulation of toxic metabolites. Newborns are not usually affected at birth since metabolites cross the placenta in utero and are metabolized by the mother [7]. Ketoacidosis with hyperammonemia usually suggests an underlying organic aciduria [9].

- Amino acid metabolism disorders: maple syrup urine disease (MSUD), nonketotic hyperglycinemia (NKH), phenylketonuria (PKU), etc.;
- Organic acid disorders: isovaleric acidemia, glutaric aciduria type I (GA-I), L-2-hydroxyglutaric aciduria (L2HGA), methylmalonic acidemia (MMA), multiple carboxylase deficiency, propionic acidemia, etc. [10];
- Urea cycle disorders (UCD): Deficiency of enzymes converting ammonia to urea, most common being ornithine transcarbamylase deficiency (OTCD).

### 2.2. Disorders of Biosynthesis and Breakdown of Complex Molecules (e.g., Lysosomal and Peroxisomal Disorders)

Some of these disorders may present in the neonatal period (Zellweger syndrome, neonatal adrenoleukodystrophy), but others manifest later with slow, progressive symptoms independent of food intake [7].

- Lysosomal storage disorders: α mannosidosis, Fabry disease, fucosidosis, Gaucher disease, Krabbe disease (globoid leukodystrophy), metachromatic leukodystrophy (MLD)**,** mucolipidosis, mucopolysaccharidoses (MPS)**,** Niemann Pick diseases, neuronal ceroid lipofuscinosis, sialic acid disorders, GM1 gangliosidosis, GM2 gangliosidosis (Tay-Sachs disease and Sandhoff disease), etc. [11];
- Peroxisomal disorders: X-linked adrenoleukodystrophy (ALD), Zellweger syndrome, etc.

### 2.3. Energy Production Disorders (e.g., Mitochondriopathies, Fatty Acid Oxidation Disorders, Lactic Acidosis Disorders)

These present with multisystemic symptoms particularly involving tissues with high metabolic utilization, such as the brain, heart, and skeletal muscle, due to energy failure. These disorders can be indistinguishable from HIE. Fatty acid oxidation disorders usually show absence of ketosis due to impaired ketogenesis, unlike organic acidurias [9]. They also present with acidosis, hypoglycemia, and hyperammonemia [10].

- Fatty acid oxidation disorders: carnitine cycle defects, mitochondrial β-oxidation disorders, electron transfer flavoprotein dehydrogenase deficiency (glutaric aciduria type II) [10];
- Primary lactic acidosis disorders: Kearns–Sayre syndrome (KSS), Leigh syndrome, leukoencephalopathy with brainstem and spinal cord involvement and high lactate (LBSL)**,** mitochondrial encephalopathy, lactic acidosis, and stroke-like episodes (MELAS), pyruvate dehydrogenase complex (PDHc) deficiency, succinate dehydrogenase (SDH) deficiency;
- Other: Molybdenum cofactor deficiency (MCD) and sulfite oxidase deficiency (SOD).

### 2.4. Other Disorders

- Leukodystrophies (limited to leukodystrophies related to IEMs): Canavan disease, Alexander disease, metachromatic leukodystrophy (lysosomal storage disorder), adrenoleukodystrophy (peroxisomal disorder);
- Lipid metabolism: Sjögren–Larsson syndrome (SLS) and Carnitine palmitoyltransferase 1 and 2 deficiencies);
- Metal metabolism: Menke’s Disease, Pantothenate kinase associated neurodegeneration (PANK), Wilson’s Disease;
- Miscellaneous: Aicardi–Goutières syndrome, creatine deficiency syndromes, galactosemia, congenital glycosylation disorders (CDG-1a), muscular dystrophy–dystroglycanopathy (congenital with brain and eye anomalies).

## 3. MRI and MRS for IEM Diagnosis

### 3.1. MRI

MRI is the imaging modality of choice for the evaluation of the CNS because it provides excellent soft tissue contrast and exceptional multiplanar anatomic detail. Multiple sequences are exploited to detect altered tissue properties in disease, generally improving diagnostic specificity potential when compared to computed tomography (CT) and ultrasound. For instance, MRI sequences such as diffusion weighted imaging (DWI) help characterize edema during acute episodes of encephalopathy. Although MRI protocols are best tailored to the suspected disorder and clinical question, in general, the following sequences are recommended: T1 weighted imaging (T1WI), T2 weighted imaging (T2WI), T2 fluid attenuated inversion recovery (FLAIR) (*age* > *1 year*) or proton density (PD) (*age* < *1 year*), DWI or diffusion tensor imaging (DTI), susceptibility weighted imaging (SWI with preferably with phase assessment capability), arterial spin labeling (ASL) perfusion, and for leukodystrophies, magnetization transfer T1WI [5].

Neuroimaging manifestations vary within and among IEMs, and may range from normal to diffuse, severe CNS disease depending on many factors such as type/severity of pathway defect, amount of toxic byproduct accumulation (if present), maturity of the brain at the time of insult, duration of injury, compensatory mechanisms, and timing of imaging during the disease course. Additionally, certain anatomic structures are selectively vulnerable to energy failure and toxic substrates [9,12]. Some patterns of characteristic brain involvement have been described: amino acid disorders (i.e., MSUD and NKH) predominantly involve white matter tracts; organic acid disorders usually involve deep gray matter; energy production/lactic acidosis disorders may involve both deep grey and white matter [10]. However, these patterns of involvement are nonspecific and may sometimes overlap with each other. An extensive systemic imaging approach of classifying MRI lesions has been covered in prior literature and is beyond the scope of this review [9,12,13].

From an imaging standpoint, IEMs should be considered and MRS should be added to the exam when one encounters:

- Symmetric brain disease, especially if it:
  ○ corresponds to previously described IEM patterns and/or;
  ○ is uncharacteristic of mimics such as HIE (e.g., basal ganglia involvement with thalamic sparing) and infection.
- Isolated or preferential involvement of the brainstem and/or cerebellum;
- Acute on chronic brain lesions (e.g., reduced and facilitated diffusion in different lesions);
- Chronic lesions and/or volume loss in a neonate;
- Progressive atrophy;
- Malformations with acquired brain lesions.

### 3.2. ^1^H MRS

^1^H MRS provides insight into the metabolic status of the brain at the time of imaging and often reveals diagnostic or suggestive metabolic profiles or mechanisms of certain IEMs.

A standard protocol for IEM evaluation may include single voxel spectroscopy (SVS), point resolved spectroscopy (PRESS), short echo time (TE) of 35 ms, repetition time (TR) of 1500 ms, 128 signal averages, and if possible, a longer TE acquisition (e.g., 144 ms if 1.5T or 288 ms if 3T). Voxel placement (2 × 2 × 2 cm default) location is predicated on the appearance of the brain or suspected disorder [4,5,14]. Common regions of interrogation include the cerebral deep gray nuclei and optional additional voxels over the parietal white matter or midline parietal gray matter. Chronic/inactive necrotic, hemorrhagic, and substantially calcific lesions are best avoided.

**Figure 1:**
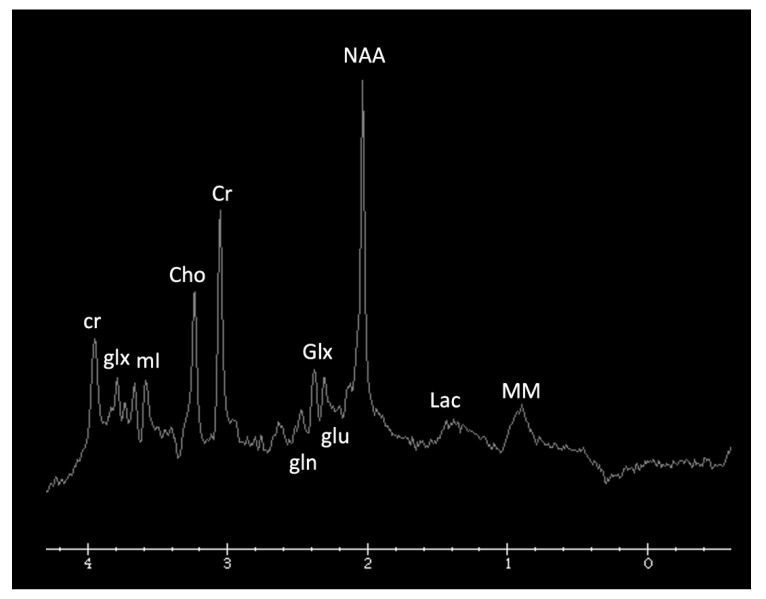
Example of a normal 3T ^1^H SVS (basal ganglia VOI) in a 3-month-old child using PRESS TE = 35 ms. Metabolic ratios change with age, with the greatest differences in the first 3 months of life. All spectra in this paper are obtained using similar parameters although voxel location is variable. Abbreviations: Cr, creatine + phosphocreatine; Cho, choline; Glu, glutamate; Gln, glutamine; Glx, glutamine + glutamate; mI, myo-inositol; MM, macromolecules; NAA, N-acetylaspartate; SVS, single voxel spectroscopy; VOI, voxel of interest.

Metabolite ratios change based on age, with the most dramatic changes in the first three months of life. Familiarity with normal age-related metabolite ratios is crucial for accurate interpretation [4,5,15]. An example short TE single-voxel (SVS) MRS performed at 3T is shown in Figure 1. The major metabolites include *N-acetylaspartate* (NAA at 2.0 ppm, neuronal metabolite and biomarker for viable neurons or assessment of parenchymal damage); *creatine* (Cr at 3.0 and 3.9 ppm, includes free creatine and phosphocreatine, marker of energetic reserve); *choline* (Cho at 3.2 ppm, marker of cellular proliferation from increased membrane turnover and/or inflammation); *myo-inositol* (mI at 3.5 ppm, glial metabolite, osmolyte, and marker of gliosis and/or neuroinflammation); *lactate* (Lac at 1.3 ppm, reflects anaerobic glycolysis); *lipid/macromolecules* (LipMM at 0.9 and 1.3 ppm from -CH3 and -CH2 groups, respectively); and *glutamate* (Glu at 2–2.5 ppm, excitatory neurotransmitter) and *glutamine* (Gln at 2–2.5 and 3.6–3.9 ppm, osmolyte and hyperammonia detoxifier) [4,5,15,16].

^1^H MRS can sometimes be diagnostic for IEMs, particularly intoxication disorders (e.g., amino acid metabolism, urea cycle disorders, and organic acid disorders) as well as disorders of biosynthesis and breakdown of complex molecules (e.g., lysosomal and peroxisomal disorders). These disorders cause accumulation of certain molecules in the brain, which manifest as characteristic peaks and/or peak patterns on spectroscopy. When lactate is sought to support a diagnosis of primary-IEM-related mitochondrial dysfunction, documenting its presence in normal appearing brain tissue is more suggestive of systemic disease than showing lactate in focal lesions with reduced diffusion, which may simply reflect local anaerobic metabolism associated with the lesion itself. Note that a small amount of lactate may be present in normal pre-term infant brains due to underactivity of pyruvate dehydrogenase, but only minimal if any lactate should be visible in term infant brains [5,15].

MRS can also help in the evaluation of leukoencephalopathies, which often present with nonspecific diffuse white matter signal changes. Leukodystrophies that present with demyelination, such as adrenoleukodystrophy (ALD), metachromatic leukodystrophy (MLD), and Krabbe disease, may show increased Cho, mI (demyelination and glial/astrocyte proliferation), decreased NAA (neuronal loss/injury), and increased Lac [13]. Other leukodystrophies such as megalencephalic leukoencephalopathy with subcortical cysts (van der Knaap disease) or vanishing white matter disease may show a diffuse decrease in metabolites [13].

## 4. MRI and/or ^1^H MRS Suggestive of IEMs (in the Appropriate Clinical Context)

**Figure 2:**
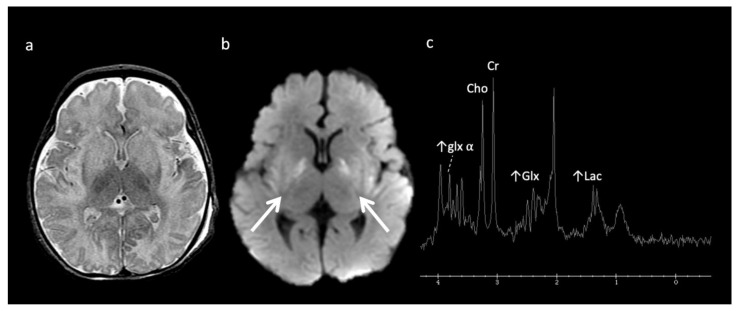
Example of hyperammonemia induced brain injury, as can be seen with urea cycle disorders. (**a**) Axial T2 and (**b**) axial diffusion weighted imaging (DWI) at the level of the basal ganglia show the typical pattern of hyperammonemic central brain involvement with perisylvian, periinsular, and basal ganglia signal hyperintensity consistent with mixed vasogenic and cytotoxic (white arrows, b) edema. (**c**) Single voxel (SV) short TE MRS demonstrates increased glutamine and glutamate (glx) with overlapping peaks at 2–2.5 ppm (2.4 ppm peak corresponds to elevated glutamine) and an elevated peak at 3.8 ppm consistent with glx associated alpha protons (glx-a). Lac and Cr are also elevated while Cho is depressed.

***Urea Cycle disorders*** (Figure 2): Urea cycle disorders (UCD) are caused by defects in the conversion of ammonia to urea, resulting in accumulation of ammonia and glutamine (Gln). Gln is osmotically active, leading to diffuse edema in the cerebral cortex and subcortical white matter when in large concentrations. Brain MR findings characteristic to UCD-related hyperammonemia include a central pattern of edema involving the peri-rolandic, peri-insular, and basal ganglia regions, often sparing the thalami, which helps distinguish it from HIE [3,7,8].

MRS shows elevated Glu/Gln peaks between 2 and 2.5 ppm during times of hyperammonemia, and a lactate doublet at 1.3 ppm when mitochondrial function fails to meet metabolic demand [3]. Glu/Gln resonances overlap at 1.5T but are more separable at 3T due to chemical shift dispersion; the peak centered at 2.4 corresponds more to glutamine [16]. There is also a commonly overlooked glx peak produced by alpha protons at 3.75 ppm. MI and Cho are usually reduced in chronic hyperammonemia, findings that can be highly suggestive of an underlying UCD in the correct clinical context [6,17].

Primary Lactic acidosis disorders: In the early stages, mitochondrial disorders with lactic acidosis may show focal edema in the deep gray nuclei, periaqueductal areas, white matter, and/or cerebellar peduncles. Later, more diffuse brain involvement may be seen [10]. Prolonged increased lactate doublet on MRS at 1.3 ppm, while nonspecific, may reflect mitochondrial encephalopathy or a problem with energy production and resultant lactic acidosis. Increased lactic acid may also be present in other IEMs such as organic acid and amino acid disorders [7]. Increased Lac seen in areas of normal brain on MRS can suggest underlying IEM [10,15].

**Figure 3:**
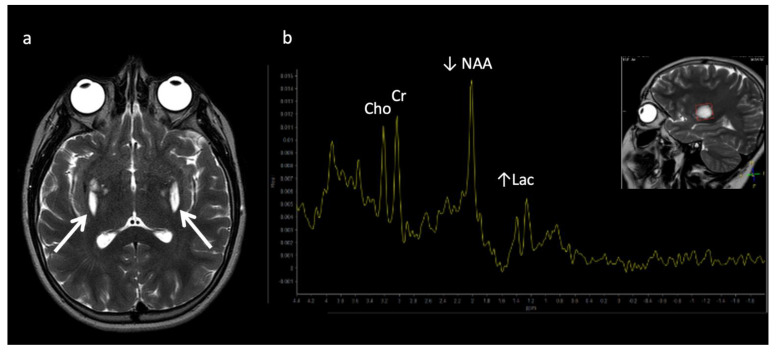
Leigh syndrome in a 10-year-old male with progressive right upper extremity weakness and left leg pain. (**a**) Axial T2WI shows T2 hyperintense necrotic lesions in the lentiform nuclei (arrows). (**b**) SV-MRS (short TE) over the basal ganglia shows increased Lac at 1.3 ppm consistent with anaerobic metabolism (indicating active on chronic disease, given the MRI appearance) and reduced NAA.

The most well-known and recognized pattern is Leigh syndrome (Figure 3) with symmetric deep grey nuclei and/or brainstem involvement on MRI [3]. Leigh syndrome may be due to a broad range of genetic variants in either nuclear or mitochondrial DNA.

Leukoencephalopathy with brainstem and spinal cord involvement with lactate elevation (LBSL), due to a defect in mitochondrial enzyme aspartyl-tRNA synthetase, is another entity, which may present with suggestive imaging findings of extensive white matter involvement concentrated in the brainstem (corticospinal, ascending sensory, pontocerebellar, and trigeminal nerve fibers) and spinal cord (lateral corticospinal tracts and dorsal columns) with variable cerebral white matter involvement (typically corticospinal tracts, corpus callosum, and other cerebral white matter sparing the U-fibers) and elevated Lac in white matter on MRS [18].

**Figure 4:**
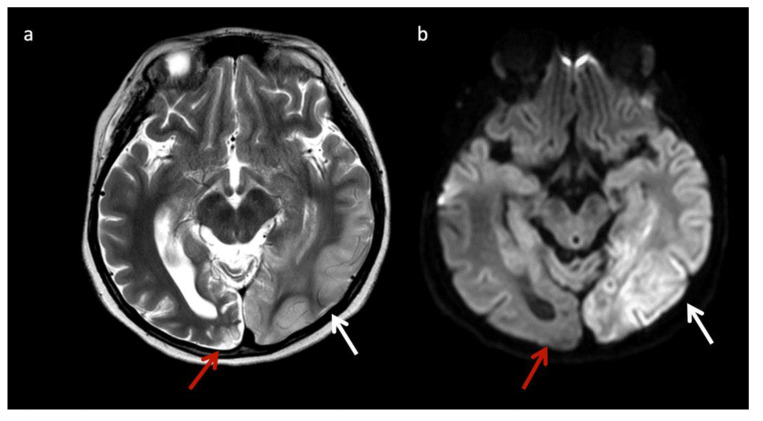
Eleven-year-old male with new onset seizures and history of multiple stroke-like episodes attributed to mitochondrial encephalopathy, lactic acidosis, and stroke-like episodes (MELAS). (**a**) Axial T2WI and (**b**) axial DWI at the level of the midbrain show nonterritorial cortical/subcortical edema/swelling in the left occipito-temporal region involving both middle and posterior cerebral artery territories consistent with acute metabolic injury (white arrows). Volume loss, T2 prolongation, and facilitated diffusion consistent with encephalomalacia from old metabolic injury is noted in the right occipito-temporal region (red arrows).

Mitochondrial encephalopathy with lactic acidosis and stroke-like episodes (MELAS) (Figure 4) may also have suggestive imaging features, with patients having non-vascular territorial metabolic stroke-like episodes [3]. Similar to MELAS, POLG-related mitochondrial disorders often cause nonterritorial cortical/subcortical edema/injury; however, perirolandic parenchyma and thalami tend to be more preferentially affected [19].

Molybdenum cofactor deficiency (MCD) and sulfite oxidase deficiency (SOD): These are disorders of sulfur-containing amino acid metabolism involved in the function of the electron transport chain. They may be indistinguishable and overlap with HIE and mitochondrial diseases both clinically and on neuroimaging. Findings that favor MCD/SOD include caudate head involvement (usually spared in HIE), thalamic sparing (often involved in HIE), chronic lesions in a neonate such as necrotic/hemorrhagic basal ganglia lesions (often asymmetric), progressive encephalopathy, facial dysmorphism, intractable seizures, high urine sulfite levels, and decreased uric acid in the serum or urine [3,7,20]. Cytotoxic edema may be present in the striatum and/or cortex/subcortical cerebral white matter with evolution to necrosis and ulegyria without preference for borderzone arterial territories (unlike HIE) [21]. MRS can demonstrate accumulated metabolites such as taurine (3.2–3.4 ppm), S-sulocysteine (3.6 ppm) and cysteine (2.9–3 ppm) as well as elevated Glu/Gln (sulfites inhibit glutamate dehydrogenase), increased Lac, and decreased NAA [7,20]. In addition, Cho tends to be elevated rather than reduced as often seen in acute HIE due to its osmolytic properties [7,22].

Biotin-thiamine responsive basal ganglia disease: SLC19A3 gene mutations cause a Leigh-like phenotype with multifocal progressive cerebral deep gray nuclear lesions (basal ganglia > thalami) that evolve to encephalomalacia, gliosis, and necrosis [23,24,25,26,27]. Cerebral cortex, white matter, brainstem, and cerebellum are less commonly involved [26,27]. MRS may show pyruvate [23].

**Figure 5:**
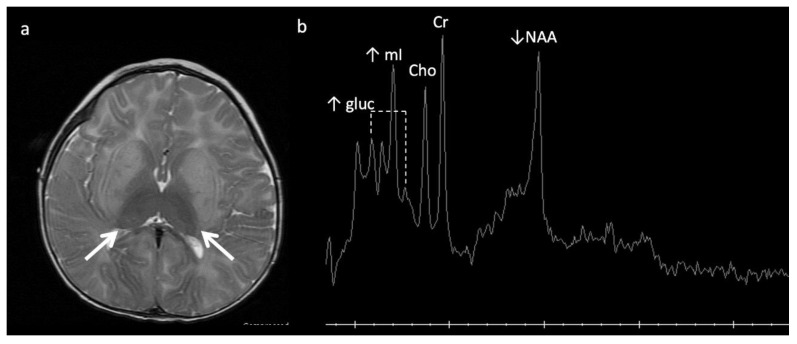
Eleven-month-old boy with Sandhoff disease (GM2 gangliosidosis), presenting with global developmental delay, hypotonia, and hyperreflexia. Axial T2WI (**a**) show abnormal hypointensity in the lateral thalami (arrows) typical of a lysosomal storage disorder, and hyperintensity consistent with edema in the bilateral basal ganglia and cerebral white matter. (**b**) SV-MRS over the basal ganglia shows reduced NAA and elevated mI—consistent with neuronal–axonal damage. Glucose (gluc peaks at 3.4, 3.8 ppm) is prominent, suggesting altered glucose (energy) metabolism.

**Figure 6:**
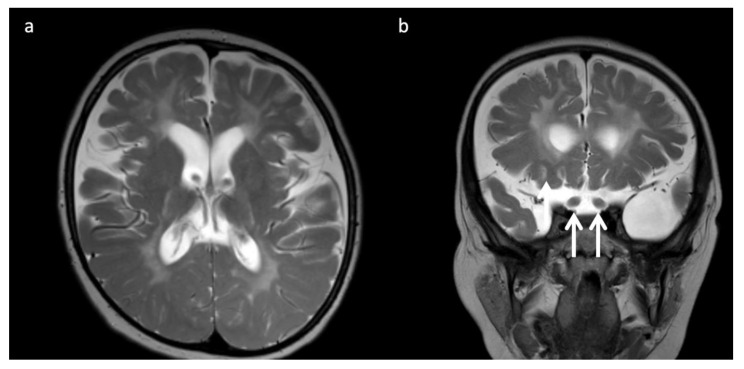
Three-year-old child with Krabbe disease and frequent seizures. Deficiency of lysosomal galactocerebroside β-galactosidase (GALC) results in accumulation of toxic psychosine. “Globoid” cells, macrophages containing galactocerebrosides, can be found in enlarged optic nerves. (**a**) Axial and (**b**) coronal T2WI show cerebral volume loss with ex vacuo ventriculomegaly, a widespread leukodystrophy with increased white matter signal sparing the U-fibers and corpus callosum, and thickening of the optic chiasm (arrows, b).

**Figure 7:**
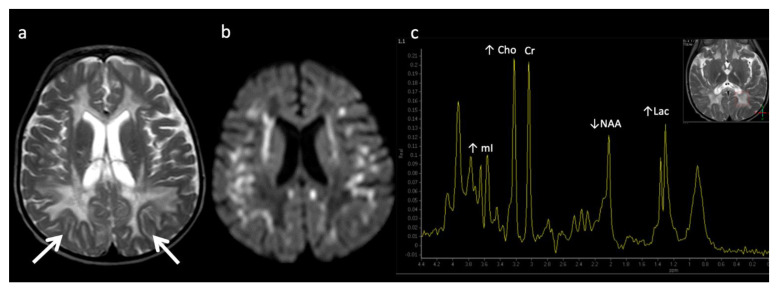
Metachromatic leukodystrophy in a 16-month-old female who presented with loss of developmental milestones. (**a**) Axial T2WI and (**b**) axial DWI demonstrate cerebral volume loss with ex vacuo ventriculomegaly, a widespread leukodystrophy with increased white matter signal sparing the U-fibers (arrows) and involving corpus callosum, and reduced diffusion at leading-edges of active demyelination. (**c**) SV-MRS over the left periatrial white matter shows elevated Lac, severely depleted NAA, and slightly elevated Cho and mI. Findings are consistent with considerable axonal damage and loss of the white matter.

Lysosomal storage disease: GM2 gangliosidoses, including Tay-Sachs disease and Sandhoff disease (Figure 5), can show characteristic T2 hypointensity in the ventral thalami and T2 hyperintensity in the basal ganglia and dorsal thalami [28]. Krabbe disease (Figure 6) may show diffuse thalamic T2 hypointensity extending to the corticospinal tracts, as well as signal abnormalities in the cerebral and cerebellar white matter, especially the dentate hila and posterior cerebral white matter (centrifugal and posteroanterior gradient often with a tigroid pattern) and variable enlargement of the optic nerve and chiasm due to accumulation of globoid cells; MR phenotypes vary with age [3,8,10,29]. Post-contrast enhancement of multiple cranial nerves and the cauda equina is also characteristic. Metachromatic leukodystrophy (Figure 7) and Krabbe disease may have overlapping imaging features; however Krabbe disease typically spares the callosal genu and more often involves the internal capsules and brainstem [30]. Neuronal ceroid lipofuscinosis may also demonstrate thalamic T2 hypointensity, with cortical atrophy as another prominent feature [9].

Aicardi–Goutières syndrome (AGS): This is an interferonopathy caused by pathogenic defects in genes that are involved in nucleotide metabolism and/or sensing [31]. Calcifications, white matter disease, and atrophy comprise the classic neuroimaging triad; however, other features correlate with the genotype, including a pseudo-TORCH neonatal presentation (TREX1), diffusely white matter signal abnormality with swelling, atrophy, and calcifications often with an infantile disease onset (RNASEH2B), bilateral striatal necrosis and subacute dystonia usually following infection with or without calcifications (ADAR1), and arterial abnormalities/vascular injury (e.g., moyamoya, aneurysms, stenosis, infarcts, and hemorrhage) (SAMHD1) [31].

Disorders of lipid metabolism: Lipid metabolism disorders such as those involving carnitine palmitoyltransferase (CPT) may have lipid elevation on MRS despite normal neuroimaging, mild brain changes, or nonspecific brain MRI abnormalities [4,6,32].

Disorders of Metal metabolism: Disorders of copper metabolism including Menke’s and Wilson’s disease may have suggestive MR imaging findings. Circle of Willis arterial tortuosity and elongation is nearly universal in Menke’s disease; white matter changes including transient vasogenic temporal white matter edema, vermian hypoplasia, progressive atrophy, and development of subdural fluid collections are additional findings [8]. These neuroimaging manifestations are especially important to recognize in young patients since the characteristic kinky hair of Menke’s disease may not be clinically apparent in the first few months of life [8]. In Wilson’s disease, brain MRI findings are age-dependent; most are normal under 10 years of age, hepatic-disease-related T1 hyperintensity may be present in the globus pallidus ± striatum and/or upper brainstem at mean age 11 years, and T2 hyperintensity may be present at mean age 13 years involving the following regions in descending order of prevalence: putamen (sometimes with central hypointensity), globus pallidus, caudate, thalamus, brainstem [33,34].

Pantothenate kinase associated neurodegeneration (PANK) is a form of neurodegeneration with brain iron accumulation (NBAI) that results in a highly suggestive MRI pattern termed “the eye-of-the-tiger sign”, which is peripheral globus pallidus hypointensity and central hypointensity on T2WI representing iron accumulation with central necrosis [35]. Clinically, extrapyramidal movement disorders develop.

## 5. MRI and/or ^1^H MRS Diagnostic Based on Disease Pattern (“Aunt Minnies”)

**Figure 8:**
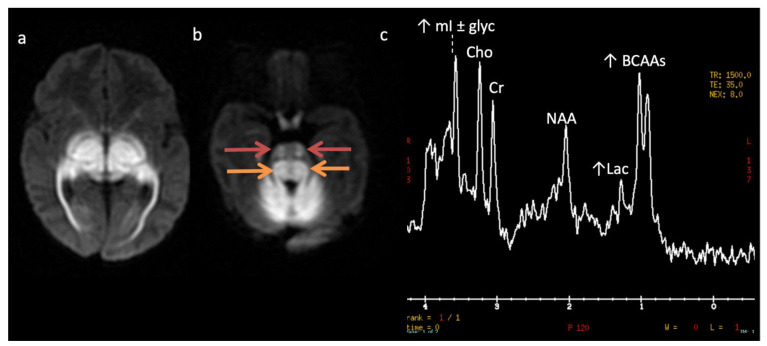
Maple syrup urine disease (MSUD) in a 23-day-old male. (**a**,**b**) Axial DWI through the level of the deep cerebrum (**a**) and pons (**b**) demonstrate diffuse symmetric markedly reduced diffusion in keeping with intramyelinic edema in the myelinated white matter tracts. Involved regions include the globi pallidi, internal capsules, thalami, and optic radiations (**a**) and the cerebellum, brainstem tracts, and optic chiasm (**b**). Note the appearance of the brainstem with involvement of the bilateral corticospinal tracts (ventral red arrows, b) and central tegmental tracts (dorsal orange arrows, b). (**c**) SV-MRS over the left basal ganglia (TR 1500, TE 35 ms) reveals a large broad doublet peak at 0.9 ppm consistent with branched chain amino- and keto-acids (BCAAs, BCKAs) and Lac. Additional findings include elevated mI and/or glycine at 3.6 ppm and mildly reduced NAA and Cho.

Maple syrup urine disease (MSUD) (Figure 8): MSUD is a rare autosomal recessive disorder caused by defective oxidative decarboxylation of the branched-chain amino acids (BCAAs) valine, isoleucine, and leucine. The accumulation of metabolites in urine leads to the odor resembling maple syrup. Characteristic MRI findings include intramyelinic edema characterized by marked diffusion restriction along myelinated white matter of the cerebrum, cerebellum, and brainstem [3,7,8]. ^1^H MRS is diagnostic, with a characteristic broadened peak complex at 0.9 ppm that inverts at intermediate TE due to branched chain amino acids and ketoacids [3]. In addition to short TE, a longer echo time MRS is useful to eliminate the overlapping peaks of lipid at 0.9 ppm. A lactate peak (anaerobic glycolysis) and decreased NAA/Cr ratio may also be present.

**Figure 9:**
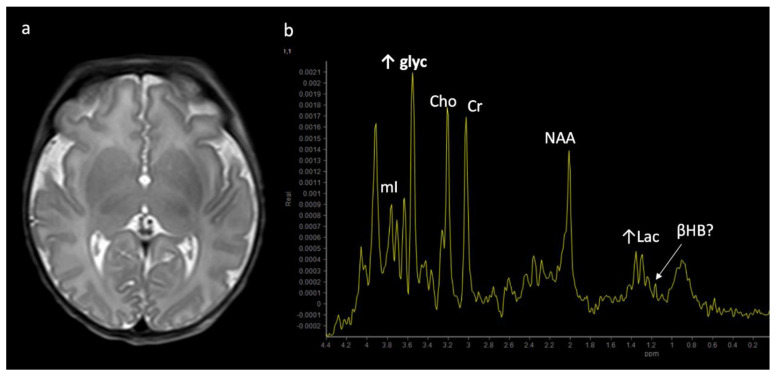
Two-week-old with seizure activity found to have nonketotic hyperglycinemia (NKH). (**a**) Axial T2WI at the level of the basal ganglia shows lack of normal myelination related hypointensity in the posterior limbs of the internal capsules. (**b**) SV-MRS over the left basal ganglia reveals a large glycine (gly) peak at 3.55 ppm (confirmed on longer TE MRS, not shown), mild Lac, mildly reduced NAA and Cho, and possible beta hydroxybutyrate (βHB) from ketosis at 1.18 ppm.

Nonketotic Hyperglycinemia (NKH) or glycine encephalopathy (Figure 9): NKH shows reduced diffusion in myelinated white matter tracts due to intramyelinic edema and vacuolization (usually involving the internal capsules, brainstem, and cerebellar white matter), with extent of involvement less prominent compared to MSUD [3,4,7,8,9]. Additional findings usually include hypogenesis of the corpus callosum and hypoplasia of the cerebellar vermis [7]. MRS reveals an elevated glycine peak at 3.55 ppm, which is best distinguished from the normal mI peak with intermediate or long echo ^1^H MRS due to its longer T2 decay [3,7,8].

Phenylketonuria (PKU): PKU is usually diagnosed at newborn screening. PKU may result in elevated phenylalanine in the brain due to deficiency of phenylalanine dehydroxylase, with a characteristic phenylalanine peak at 7.37 ppm on MRS using a short TE [3,9]. MRI may show increased T2 signal in the periventricular and subcortical white matter [3,20].

**Figure 10:**
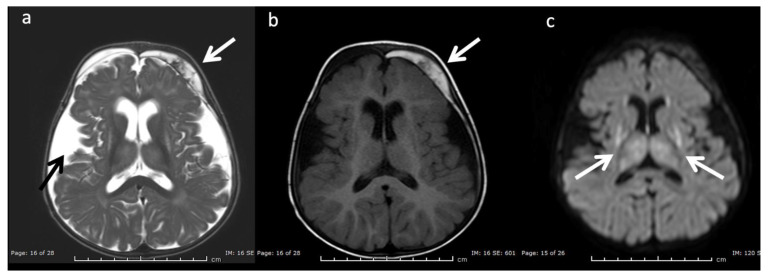
Eleven-month-old female with Glutaric aciduria Type I with acute decompensation after illness. (**a**) Axial T2WI, (**b**) T1WI, and (**c**) DWI at the level of the basal ganglia demonstrate diffuse cerebral volume loss, underopercularization (black arrow, a), bifrontal chronic subdural hemorrhages with superimposed subacute left frontal subdural blood products (white arrows, a and b), and reduced diffusion in the lentiform nuclei and thalami (white arrows, c).

Glutaric Aciduria Type 1 (GA-I) (Figure 10): GA-I is a disorder of lysine, hydroxylysine, and tryptophan catabolism that results in characteristic MRI findings of poorly formed operculum, widened Sylvian fissures and frontotemporal CSF spaces, large cavum septi pellucidi, and basal ganglia lesions [3,7,8]. Supratentorial subdural hematomas may develop over time as a consequence of cerebral atrophy [20,36]. GA-I should be distinguished from glutaric aciduria type II that is caused by the inability to breakdown proteins and fats for energy and may present with underdeveloped frontotemporal lobes and enlarged sylvian fissures, delayed myelination, and hypoplasia of the corpus callosum [37].

L-2-hydroxyglutaric aciduria (L2HGA): In this disease, there is an accumulation of L-2-hydroxyglutaric acid due to a mitochondrial enzyme gene L2HGDH mutation. MRI typically shows a centropedal pattern of brain involvement with edema within the frontal and subcortical white matter, which progressively becomes more confluent but spares the brainstem. The dentate nuclei and basal ganglia are usually involved, but the thalami are spared [20,38]. Another IEM with centrifugal white matter involvement, Kearns–Sayre syndrome, typically shows calcifications unlike L2HGA. In L2HGA, MRS may reveal decreased NAA and increased mI.

**Figure 11:**
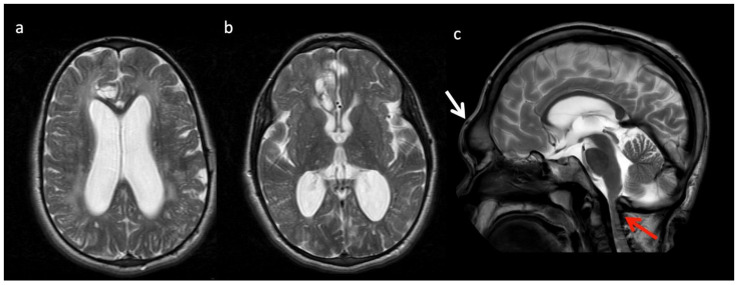
Thirteen-year-old male with Hurler’s disease (mucopolysaccharidosis). (**a**,**b**) Axial T2W at the level of the corona radiata (**a**) and third ventricle (**b**) show diffuse white matter hyperintensity, multiple enlarged perivascular spaces, and generalized ventriculomegaly. Volume loss in the right frontal lobe may be due to prior injury. (**c**) Sagittal T2 images from a different 13-year-old male with Hurler’s shows frontal bossing (white arrow), dens hypoplasia, platyspondyly, J-shaped sella, and thickened dural ring at the foramen magnum with craniocervical junction (CVJ) stenosis (red arrow).

Mucopolysaccharidoses (MPS) (Figure 11): MPS are a group of lysosomal storage disorders that demonstrate characteristic though inconstant MR features of enlarged perivascular spaces, cerebral white matter hyperintensity on T2/FLAIR, and ventriculomegaly [8]. Spinal imaging shows a dysostosis multiplex. MRS can demonstrate elevated Cho (gliosis and demyelination) and peaks at 3.6–3.7 ppm from mucopolysaccharides accumulated in the brain [3].

α-Mannosidosis: MRS shows a broadened peak (3.5–3.9 ppm) representing mannose-rich oligosaccharides that can resolve following bone marrow transplant. On MRI, hypomyelination and leukodystrophy are present.

Fucosidosis: MRS may show a broadened peak at 3.8–3.9 ppm attributed to carbohydrate-containing macromolecules, such as mannosidosis. However, there is an additional peak at 1.2 ppm that inverts at intermediate echo time attributable to fructose, which makes the diagnosis [39]. Characteristic MRI abnormalities include hypomyelination with T1 and T2 shortening in the globus pallidus, T2 prolongation in the globus pallidus internal medullary lamina, and callosal thinning [40].

Salla disease: This is a lysosomal disorder causing a defect in sialic acid transport, resulting in elevated N-acetyl neuraminic acid. An elevated peak of the N-acetyl methyl group at 2.0 ppm on MRS may be confused with NAA [41]. This is rare and usually seen in Scandinavian ancestry, with minimal or slow myelination, cerebral subcortical white matter involvement sparing the deep white matter, accelerated iron deposition most pronounced in the globus pallidus, thinning of the corpus callosum, and variable cerebellar atrophy [8,40,41].

**Figure 12:**
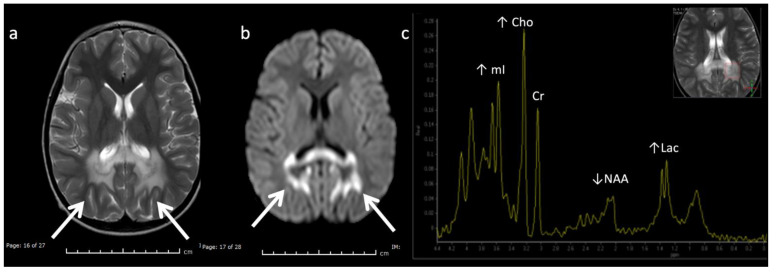
Six-year-old male with X-linked Adrenoleukodystrophy (ALD). (**a**) Axial T2 and (**b**) DWI images reveal confluent T2 hyperintensity (**a**) and reduced diffusion (**b**) involving the callosal splenium and forceps major/peri-trigonal white matter (white arrows) with sparing of subcortical U-fibers. The pattern of involvement has a postero-anterior and centrifugal pattern. A zonal pattern of signal alteration with mixed diffusion abnormalities indicates acute on chronic demyelination related injury. (**c**) SV-MRS of the left periatrial white matter reveals decreased NAA (neuronal loss, decreased neuronal-axonal integrity, and/or decreased production), elevated Cho (increased membrane turnover), increased mI (neuroinflammation marker), and increased Lac (anaerobic metabolism).

X-linked Adrenoleukodystrophy (ALD) (Figure 12): This peroxisomal disorder is due to a defect in oxidation of long-chain fatty acids resulting in their accumulation. Lesions usually initiate in the callosal splenium and spread into the forceps major, projectional fibers, and auditory and visual pathway; however, in a minority of cases, they may begin in the callosal genu and extend into the forceps minor and beyond [3,41]. Laminated zones of signal alteration in the involved cerebral areas are characteristic, with reduced diffusion and post-contrast enhancement during active demyelination and inflammation, The addition of X-ALD to newborn screen testing has brought about pre-symptomatic MR screening; these scans require careful scrutiny for early/mild changes with special attention to the corpus callosum [42]. Boys with X-ALD should be monitored with serial MRIs based on consensus guidelines [43]. MRS demonstrates decreased NAA and elevated Cho and mI, findings that can improve after successful stem cell transplant [44,45].

**Figure 13:**
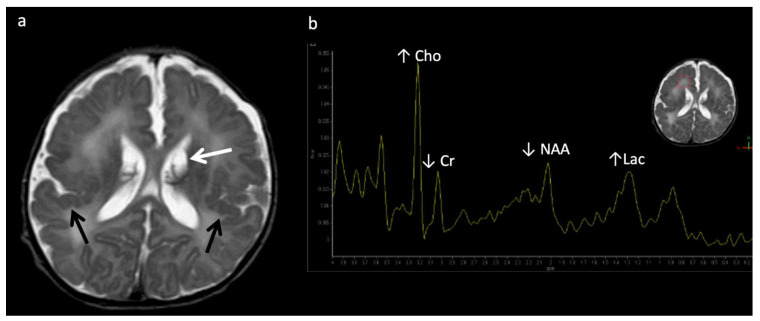
Zellweger syndrome in a 4-day-old full-term infant with multiple congenital anomalies, hypotonia, and weak/absent reflexes. (**a**) Axial T2WI shows findings typical of Zellweger syndrome, including bilateral perisylvian polymicrogyria (black arrows), germinolytic cysts (white arrow), and abnormal white matter, which may represent areas of hypomyelination. (**b**) SV-MRS of the white matter shows elevated Lac (consistent with anaerobic metabolism and indicating active on chronic disease, given MRI appearance), elevated Cho, and depleted NAA and Cr (compatible with neuronal/axonal damage/loss).

Zellweger syndrome (Figure 13): Peroxisomal function is vital to neuronal migration and organization and myelination [9]. PEX gene defects account for most peroxisomal bioassembly disorders, including the milder phenotype, peroxisome biogenesis disorder-1B (PBD1B) comprising neonatal adrenoleukodystrophy and infantile refsum disease. Characteristic MRI findings of Zellweger syndrome include cortical malformations, germinolytic cysts, white matter abnormalities, and reduced gray and white matter volume [3,7,8]. D-bifunctional protein deficiency caused by a disorder of peroxisomal fatty acid beta-oxidation may manifest similar findings. PBD1B also may have overlapping neuroimaging abnormalities, but lacks the systemic findings (e.g., renal cysts, chondrodysplasia punctata) seen in Zellweger syndrome [9]. Dentate hilar/superior cerebellar peduncle involvement progressing to involve the cerebellar white matter more diffusely, the brainstem, thalami, and cerebrum with a posteroanterior gradient typifies the temporal pattern of PBD1B [46]. MRS may show lipid elevation and findings secondary to hepatocellular dysfunction (increased Glu and Gln, decreased mI) [3,8].

**Figure 14:**
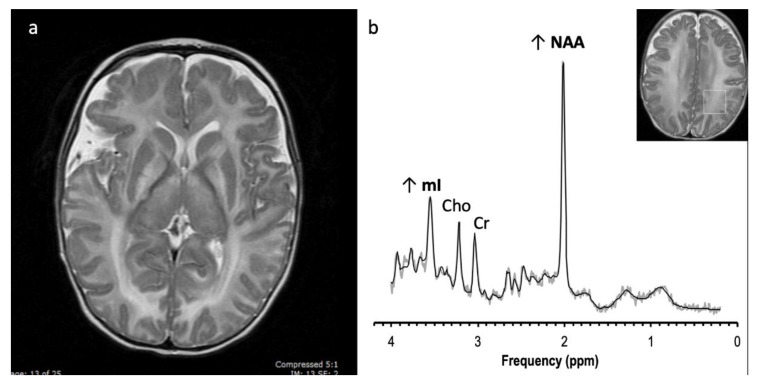
Canavan disease in a 4-month-old infant who presented with hypotonia, spasticity, and elevated urine organic acids. (**a**) Axial T2WI reveals diffuse white matter hyperintensity involving the subcortical U fibers throughout, the globi pallidi, and thalami with sparing of the striatum. (**b**) SV-MRS of the left parietal white matter shows severely elevated NAA (210–240% above normal) due to lack of enzyme degradation. MI is also elevated, and Cho and Cr are low.

**Canavan disease** (Figure 14): Canavan disease is due to a deficiency of aspartoacylase, which catalyzes the hydrolysis of NAA and leads to accumulation of NAA [20]. Macrocephaly is typically present but not universal. On MRI, diffuse spongiform changes are present involving the white matter, thalami and globi pallidi but sparing the caudate nuclei and putamina. Reduced diffusion is found in the involved white matter during the active phase of disease. ^1^H MR spectra is pathognomonic with a markedly increased NAA peak at 2.01 ppm.

**Figure 15:**
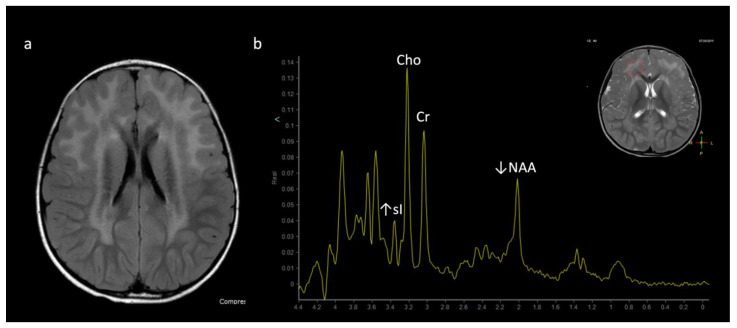
Alexander disease in a 3-year-old female with dysmorphic features and developmental delay. (**a**) Axial T2 FLAIR image demonstrates widespread hyperintense white matter signal involving the subcortical U fibers only in the frontal regions consistent with a leukodystrophy with an anteroposterior severity gradient. (**b**) SV-MRS of the right frontal white matter shows slightly elevated Lac, reduced NAA, and elevated scyllo-inositol at 3.36.

**Alexander disease** (Figure 15): Alexander disease is an astrocytopathy, which similar to Canavan disease, may present as a macrocephalic leukodystrophy. Frontal disease predominance, striatal involvement, thalamic sparing, post-contrast enhancement and lack of restricted diffusion usually distinguish Alexander from Canavan disease [3]. MRS generally shows elevated inositols, and unlike Canavan disease, reduced NAA [3].

**Figure 16:**
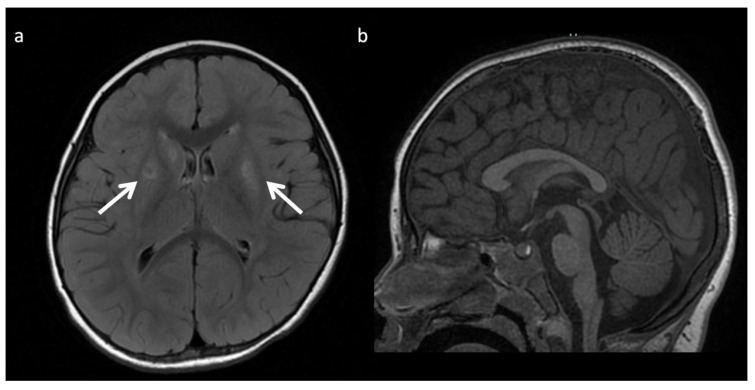
Pyruvate dehydrogenase complex (PDHc) deficiency in a 30-month-old female with desaturations. (**a**) Axial T2 FLAIR image shows a Leigh pattern of brain injury with bilateral putamen and right caudate head (white arrows) hyperintense lesions with central signal suppression representing necrosis. (**b**) Sagittal T1WI shows mild pontine hypoplasia and thinning of the callosal splenium, likely representing regional hypogenesis rather than volume loss, given low normal callosal length and normal cerebral white matter depth.

Pyruvate dehydrogenase complex (PDHc) deficiency (Figure 16): PDHc deficiency is due to impaired pyruvate to acetyl-coA conversion and lactate accumulation. MRS shows elevated Lac and pyruvate at 2.37 [8]. There are two distinct PDHc deficiency phenotypes, (1) prenatal onset with destructive changes and brain malformations such as dysgenesis of the corpus callosum and neuronal migrational abnormalities, and (2) postnatal onset energy failure with Leigh disease [3,8].

Succinate dehydrogenase (SDH) deficiency: Absent or insufficient oxidation of succinate to fumarate and electron delivery to the respiratory chain results in significantly elevated Lac levels and a specific succinate peak that can be detected at 2.4 ppm in affected white matter [47]. MRI shows involvement of the cerebral white matter (sparing the U-fibers and corpus callosum outer fibers), corticospinal tracts, middle cerebellar peduncles, spinal cord, and specific thalamic regions [47].

**Figure 17:**
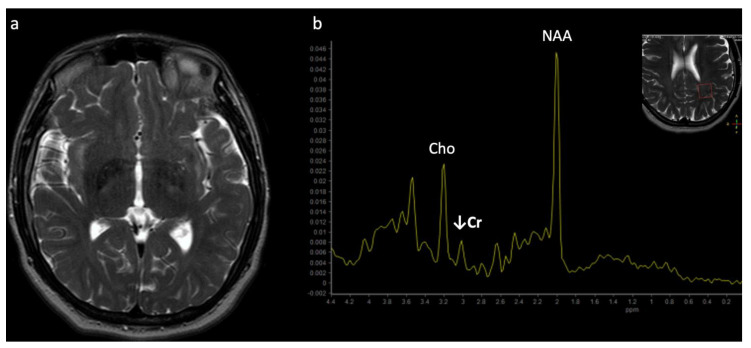
Twenty-year-old male with epilepsy and learning disability, with X-linked creatine transporter deficiency. (**a**) Axial T2WI of the brain is unremarkable. (**b**) SV short TE MRS over the left parietal white matter reveals Cr levels significantly below normal (~10–20% of normal at 3 and 3.9 ppm). NAA and Cho within normal limits.

Creatine deficiency syndromes (Figure 17): These include disorders of biosynthesis and transport of creatine, including guanidinoacetate methyltransferase deficiency (GAMT gene) [48] and L-arginine-glycine amidinotransferase deficiency (GATM gene) [49], and creatine transporter deficiencies (an X-linked disorder with SLC6A8 gene mutations) [17,50]. Creatine is essential for neuronal energy storage and transmission [15]. MRI is usually either normal or shows mild, nonspecific changes such as volume loss. However, ^1^H MRS shows the diagnostic markedly reduced or completely absent Cr peaks at 3 and 3.9 ppm [3,6,17]. An abnormal broad guanidinoacetate peak is present in patients with GAMT gene defects at 3.78 ppm [7].

Galactosemia: Galactosemia is due to a deficiency of galactose-1-phosphate enzyme and results in the accumulation of galactose-1-phosphate and galactitol. A galactitol peak at 3.7 ppm (doublet at short TE; peak inversion at intermediate TE) and reduced mI are characteristic on MRS [6,9]. MRI may be normal or show nonspecific abnormalities such as multifocal or confluent frontoparietal white matter lesions to diffuse brain edema [51].

**Figure 18:**
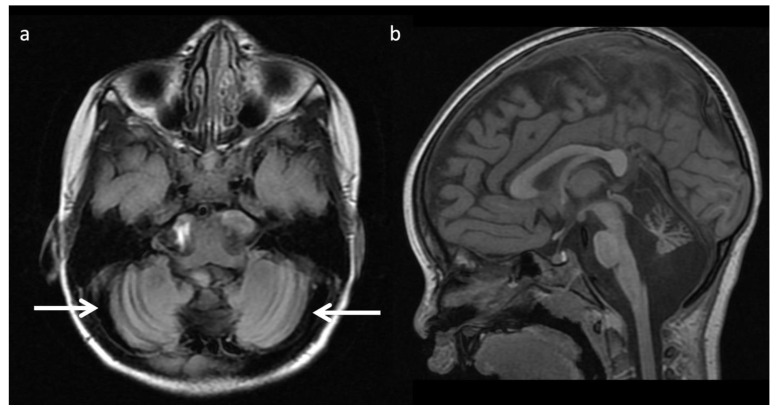
Three year old child with congenital disorder of glycosylation Type 1a (CDG-1a), which is an early-onset neurodegenerative disorder with selective hindbrain involvement. (**a**) Axial T2 FLAIR image of the cerebellum shows diffuse marked cerebellar atrophy and diffuse cerebellar hyperintense T2 signal (white arrow). (**b**) Sagittal T1 image again shows the marked cerebellar atrophy. The pons is also mildly hypoplastic.

Congenital disorder of glycosylation Type 1a (CDG-1a) (Figure 18): CDGs are genetically heterogenous autosomal disorders caused by abnormal glycosylation of N-linked oligosaccharides. CDG-1a is the most common form and is an early onset neurodegenerative disorder with selective hindbrain involvement and variable clinical presentation. Key MR findings are diffuse cerebellar volume loss with diffuse cerebellar T2/FLAIR hyperintense signal [52,53]. Other findings include progressive volume loss of the cerebellum and pons, as well as the supratentorial white matter [52]. MRS findings include reduced NAA/Cr ratios and increased mI [52].

Muscular dystrophy–dystroglycanopathy (congenital with brain and eye anomalies): This is a heterogenous group of neuromuscular disorders due to reduced glycosylation of alpha-dystroglycan with somewhat poor phenotype–genotype correlation. Characteristic neuroimaging findings include extensive malformations of cortical development of various types, especially cobblestone lissencephaly, white matter disease, hydrocephalus, and brainstem and variable cerebellar hypoplasia/dysgenesis [8]. Peripheral cystic appearing areas along the cerebellar surface are typical findings but may not be visible without high resolution sequences. Peculiar brainstem deformities are often present in more severe forms, including a kinked, z-shape brainstem, midline pontine clefting, and bulbous appearance of the midbrain, among others [8]. Ocular abnormalities are variable but frequent; although a formal ophthalmologic assessment may be necessary for detection, many are visible on brain MRI, for instance persistent fetal vasculature and microphthalmia.

## 6. Mimics of IEMs and Utility of MRI and/or ^1^H MRS to Support or Refute Diagnosis

HIE, infection, trauma, demyelinating disorders, and toxic-acquired metabolic disorders can mimic IEMs and should be considered depending on the clinical circumstances. A brief review of common imaging patterns in HIE and CNS infection is warranted since these are statistically more common and always need to be excluded. In the future, there may be more sophisticated algorithms available to better diagnosis IEMs and to distinguish IEMs from other differentials [54]. However, clinical history is still crucial for making an accurate diagnosis.

Clinically, IEMs present with progressive deterioration after normal prenatal course/birth/delivery, prolonged instability, or progression, in contrast to HIE, which manifests within a few hours after birth and tends to stabilize by 2–3 weeks [55]. HIE is the most common cause of neonatal encephalopathy and HIE injury patterns depend primarily on the degree of brain development at the time of injury, severity, and duration of injury [55]. Typical patterns include borderzone arterial territory injury in partial prolonged ischemia, basal ganglia/thalamic involvement in profound ischemia, and in the most severe circumstances, global brain injury when the brain is matured to term and beyond [55]. In IEMs such as urea cycle disorders, the thalami may be spared, which is unlike HIE. MRS abnormalities including NAA reduction, abnormal Lac, and Glu elevation correlate with injury severity and help predict prognosis but do not distinguish HIE from an underlying IEM. Creatine concentrations changes are common; creatine is often elevated in milder forms of HIE due to altered ATP utilization, changing the ratios of NAA:Cr and Cho:Cr.

CNS infections may involve grey and/or white matter in a patchier, often more asymmetric distribution compared to IEMs and without large arterial territory confinement. However, some acute necrotizing encephalitis (ANE) infections can involve the deep gray nuclei diffusely and symmetrically similar to HIE and IEMs (e.g., organic acidurias or mitochondrial disorders). Congenital TORCH infections (such as toxoplasmosis and cytomegalovirus) may result in calcifications, microcephaly, and, in CMV, migrational disorders [28]. Likewise, para-infectious or autoimmune demyelinating disease such as acute disseminated encephalomyelitis (ADEM) may involve the white matter, basal ganglia, and thalami, but lesions in these areas are usually asymmetric, white matter is typically involved, and the clinical course is usually not progressive (typically monophasic illness) [55,56]. A rim-like region of increased T2 signal along the edge of involved basal ganglia has been reported in ADEM and ANE [54].

Traumatic brain injury may also result in cerebral restricted diffusion in the neonate, which may be seen with HIE and IEMs. Evaluation for any history of trauma must be considered.

Finally, toxic-acquired metabolic disturbances may also cause similar MRI findings found with IEMs. Carbon monoxide and drugs such as heroin and MDMA can cause infarcts of the bilateral globi pallidi, which are also seen with disorders such as methylmalonic acidemia and PDHc deficiency [9,12]. Wernicke encephalopathy (thiamine vitamin B1 deficiency) can cause signal changes in the thalami, putamina, tectal plate, and periaqueductal grey matter similar to Leigh disease; however, hypothalamic/mamillary body involvement favors Wernicke’s encephalopathy [56,57]. Extrapontine osmotic myelinolysis (rapid correction of hyponatremia) can result in symmetric thalamic and basal ganglia abnormalities due to destruction of myelin and edema [56]. Vigabatrin treatment (GABA inhibitor) used in infantile spasms may result in hyperintense T2 signal and restricted diffusion involving the thalami, globus pallidus, anterior commissure fibers, central tegmental tracts, dentate nucleus, and cerebral peduncles [28,57]. These findings can be confused with IEMs but can be differentiated by clinical history and pattern of involvement.

## 7. Conclusions

Neuroimaging plays a critical role in the diagnostic evaluation of IEMs. Radiological and/or clinical phenotyping may rarely suggest a particular entity, which can allow for single gene testing. More often, radiological phenotyping of IEMs may point to a specific disease category, prompting a particular gene panel analysis. With metabolic profiling, the phenotype is often nonspecific but can allow for whole exome sequencing [9]. If the work-up is unrevealing, whole genome sequencing, chromosomal microarray, or other workup may be required for further evaluation [9].

IEMs are a heterogenous group of disorders that are difficult to diagnosis, given their often nonspecific clinical and imaging presentations. However, many have characteristic neuroimaging and MRS patterns. This paper reviews and clarifies the role of MRI and ^1^H MRS in the evaluation of IEMs and focuses on scenarios where they are suggestive of or diagnostic for IEMs.

## Data Availability

Not applicable.

## Appendix A

**Table A1:** Inborn errors of metabolism with unique MRS and MRI profiles that can be suggestive or diagnostic *.

Disorder	Classification	Defect + Metabolic Consequence	Key MRS Metabolite (ppm) or MRI Feature
Maple syrup urine disease (MSUD) *	Amino aciduria	Defect in branched-chain keto-acid dehydrogenase enzyme ➔ ↑ branched chain amino acids and ketoacids (BCAAs, BCKAs)	↑ BCAAs + BCKAs (0.9) Intramyelinic edema involving cerebrum, cerebellum, brainstem
Non-ketotic hyperglycinemia (NKH) *	Amino aciduria	Defective mitochondrial enzyme involved in glycine cleavage ➔ ↑ glycine	↑ Glycine (3.5) Intramyelinic edema, Hypogenesis corpus callosum, vermian hypoplasia
Phenylketonuria (PKU) *	Amino aciduria	Phenylalanine hydroxylase deficiency	↑ Phenylalanine (7.37) Periventricular and subcortical white matter (WM) abnormalities
Glutaric Aciduria type I (GA-I) *	Organic aciduria	Enzyme deficiency altering lysine, hydroxylysine, tryptophan metabolism➔ ↑ glutaric acid, hypoglycemia	Poorly formed operculum, widened sylvian fissures and frontotemporal subarachnoid spaces, basal ganglia (BG) lesions
L-2-hydroxyglutaric aciduria (L2HGA) *	Organic aciduria	Mitochondrial enzyme L2HGDH mutation➔ ↑ L-2-hydroxyglutaric acid	Initial frontal and subcortical WM, with later confluent WM and BG abnormality. Dentate nuclei lesions
Methylmalonic acidemia (MMA)	Organic aciduria	Defect in methylmalonyl- coenzyme A mutase➔ ↑ methylmalonic acid, glycine, ammonia	Cerebral WM and globus pallidus lesions
Propionic acidemia	Organic aciduria	Defect in propionyl-coenzyme A carboxylase	↑ propionic acid, glycine Cerebral WM and striatum lesions
Urea cycle defects (UCD)	Urea cycle defects (UCD)	Deficiency in detoxification of ammonia to urea➔ ↑ ammonia, glutamine	↑ Glu ± ↓ mI and Cho Cortical and subcortical lesions usually sparing thalamus
α-Mannosidosis *	Lysosomal	Deficiency of α-mannosidase	Mannose-rich oligosaccharides (3.5–3.9) Hypomyelination Leukodystrophy (LD)
Fucosidosis *	Lysosomal	Deficiency of α-L-fucosidase needed to metabolize fucose-containing compounds	Carbohydrate-containing macromolecules (3.8–3.9) Fructose (1.2 doublet); inverts at intermediate echo time Hypomyelination, thalamic and GP T2 hypointensity
Globoid cell leukodystrophy (Krabbe disease)	Lysosomal	Galactocerebroside β-galactosidase deficiency ➔ globoid cell accumulation	Thalamic T2 hypointensity Centrifugal gradient LD, tigroid WM pattern, cranial nerve enhancement, optic nerve enlargement
Metachromatic leukodystrophy (MLD)	Lysosomal	Decreased arylsulfatase A enzyme activity ➔ metachromatic sulfatide deposits	Centrifugal gradient LD, tigroid WM pattern, cranial nerve enhancement
Mucopolysaccharidosis (MPS) *	Lysosomal	Deficiencies in lysosomal hydrolases responsible for metabolizing mucopolysaccharides (a.k.a. glycosaminoglycans)	Mucopolysaccharides (3.6–3.7) ↑ Cho Enlarged perivascular spaces, ventriculomegaly, WM lesions, dysostosis multiplex, CVJ stenosis
Salla disease *	Lysosomal	Defect in sialic acid transport ➔ N-acetyl neuraminic acid	↑ N-acetyl neuraminic acid (2) Diffuse WM abnormality
Tay-Sachs and Sandhoff (GM-2 gangliosidosis)	Lysosomal	Reduced beta-hexosaminidase enzyme➔ ↑ GM2-ganglioside accumulation	Sandhoff (N-acetylhexosamine metabolite at 2.1) Thalamic T2 hypointensity Striatum T2 hyperintensity
X-linked adrenoleukodystrophy (ALD) *	Peroxisomal	Inability to oxidize long-chain fatty acids (VLCFA) into short-chain fatty acids ➔ accumulation of long-chain fatty acids	Peri-trigonal T2 hyperintensity and restricted diffusion Posteroanterior & centrifugal gradient
Zellweger syndrome *	Peroxisomal	Decreased dihydroxyacetone phosphate acyl transferase (DHAP-AT) activity. Peroxisomal function crucial to neuronal migration.	Lipids (0.87, 1.27) peri-sylvian polymicrogyria, germinolytic cysts
Biotin-thiamine responsive basal ganglia disease	Thiamine metabolism	Mutation in SCL19A3 gene encoding a thiamine transporter	Leigh-like phenotype Pyruvate (2.37)
Leigh disease (subacute necrotizing encephalopathy)	Mitochondrial	Multiple mutations in mitochondrial or nuclear DNA	↑ Lac (1.33) Pattern of symmetric basal ganglia or brainstem abnormalities
Leukoencephalopathy with brainstem and spinal cord involvement (LBSL)	Mitochondrial	Mitochondrial aspartyl-tRNA synthetase deficiency	↑ Lac (1.33), mI, Cho, ↓ NAA Diffuse cerebral volume loss, involvement of brain and spine
Mitochondrial encephalopathy, lactic acidosis, and stroke-like episodes (MELAS) and POLG-related mitochondrial disorders	Mitochondrial	Mutations in mitochondrial DNA	↑ Lac (1.33) Non-territorial and basal ganglia “stroke-like” lesions Peri-rolandic parenchyma and thalami preferentially affected in POLG-related disorders
Pyruvate dehydrogenase complex (PDHc) deficiency *	Mitochondrial	Impaired pyruvate to acetyl-coA conversion and lactate accumulation	Pyruvate (2.37), ↑ Lac Leigh disease pattern Germinolytic cysts Periventricular necrosis
Molybdenum cofactor deficiency (MCD) and Sulfite Oxidase Deficiency (SOD)	Amino aciduria/Electron transport chain	Defect in amino acid metabolism, involved in electron transport chain	↑ taurine (3.2–3.4), ↑ S-sulocysteine (3.6), ↑ cysteine (2.9–3), ↑ Glx, ↑ Lac, ↑ Cho, ↓ NAA. Caudate head involved, thalamic sparing.
Succinate dehydrogenase (SDH) deficiency *	Mitochondrial	Absent/insufficient oxidation of succinate ➔ fumarate and electron delivery to the respiratory chain	Succinate (2.4), ↑ Lac (1.33) Leigh disease pattern
Alexander disease *	Leukodystrophy (Macrocephalic)	Astrocytopathy resulting in defect in myelin deposition	Frontal predominant WM disease and striatum involvement, enhancement, ↑ mI and sI
Canavan disease *	Leukodystrophy (Macrocephalic)	Inability to metabolize N-acetyl asparate (NAA) into asparate and acetate	↑↑ NAA Diffuse WM and thalamic lesions sparing striatum
Menke’s Disease	Metal Metabolism	Copper metabolism Defect	Circle of Willis tortuosity and elongation universal, ± WM changes, vermian hypoplasia, atrophy, subdural collections
Pantothenate kinase associated neuro- degeneration (PANK)	Metal Metabolism	Neurodegeneration with brain iron accumulation	“Eye-of-the-tiger” sign—peripheral and central globus pallidus T2 hypointensity
Wilson’s Disease	Metal Metabolism	Copper metabolism Defect	T1 hyperintensity in globus pallidus ± striatum and/or upper brainstem (11 years) T2 hyperintensity in putamen, globus pallidus, caudate, thalamus, brainstem (13 years)
Aicardi–Goutières syndrome	Miscellaneous	Defect in genes involved in nucleotide metabolism and/or sensing	Classic Triad: Calcifications, WM disease, atrophy Various other features correlate with genotype
Carnitine palmitoyltransferase (CPT)	Miscellaneous	Disorder of lipid metabolism	↑↑ Lipid
Creatine deficiency disorders *	Miscellaneous	Disorders of biosynthesis and transport of creatine	Reduced or absent Cr (3) MRI may be normal
Galactosemia *	Miscellaneous	Deficiency of galactose-1-phosphate enzyme ➔ ↑galactose-1-phosphate and galactitol	Galactitol (3.7): doublet at short TE, peak inversion at intermediate TE; ↓ mI
Congenital disorder of glycosylation Type 1a (CDG-1a)	Miscellaneous	Mutation in gene encoding PMM2 ➔ abnormal glycosylation of N-linked oligosaccharides	Marked cerebellar volume loss with diffuse cerebellar T2 hyperintensity. Progressive volume loss of pons, cerebellum, and supratentorial WM. ↓ NAA/Cr ratio, ↑mI
Muscular dystrophy- dystroglycanopathy (congenital with brain and eye anomalies) *	Miscellaneous	Reduced glycosylation of Alpha-dystroglycan	Extensive malformations of cortical developmental (i.e., cobblestone lissencephaly, kinked z-shaped brainstem, midline pontine clefting)
